# Breast cancer biological subtypes and protein expression predict for the preferential distant metastasis sites: a nationwide cohort study

**DOI:** 10.1186/bcr2944

**Published:** 2011-09-13

**Authors:** Harri Sihto, Johan Lundin, Mikael Lundin, Tiina Lehtimäki, Ari Ristimäki, Kaija Holli, Liisa Sailas, Vesa Kataja, Taina Turpeenniemi-Hujanen, Jorma Isola, Päivi Heikkilä, Heikki Joensuu

**Affiliations:** 1 Laboratory of Molecular Oncology, University of Helsinki, Biomedicum Helsinki, Haartmaninkatu 8, 00290, Helsinki, Finland; 2 Department of Oncology, Helsinki University Central Hospital and University of Helsinki, Haartmaninkatu 4, 00029 HUS, Helsinki, Finland; 3 Institute for Molecular Medicine Finland, University of Helsinki, P.O. Box 20, 00014, Helsinki, Finland; 4 Division of Global Health/IHCAR, Karolinska Institutet, Nobels väg 9, 171 77 Stockholm, Sweden; 5 Department of Pathology, HUSLAB, Helsinki University Central Hospital, Haartmaninkatu 3, 00029 HUS, Helsinki, Finland; 6 Haartman Institute and Research Program Unit, Biomedicum Helsinki, Haartmaninkatu 8, University of Helsinki, 00014 Helsinki, Finland; 7 Department of Oncology, Tampere University Hospital, Teiskontie 35, 33014, Tampere, Finland; 8 Department of Oncology, Turku University Central Hospital, Hämeentie 11, 20521, Turku, Finland; 9 Department of Oncology, Kuopio University Hospital, Puijonlaaksontie 2, 70211, Kuopio, Finland; 10 Department of Oncology, Vaasa Central Hospital, Hietalahdenkatu 2-4, 65000, Vaasa, Finland; 11 Department of Oncology and Radiotherapy, Oulu University Hospital, Kajaanintie 50, 90220 Oulu, Finland; 12 Laboratory of Cancer Biology, Institute of Medical Technology, University of Tampere and Tampere University Hospital, Biokatu 8, 33520, Tampere, Finland

## Abstract

**Introduction:**

Some molecular subtypes of breast cancer have preferential sites of distant relapse. The protein expression pattern of the primary tumor may influence the first distant metastasis site.

**Methods:**

We identified from the files of the Finnish Cancer Registry patients diagnosed with breast cancer in five geographical regions Finland in 1991-1992, reviewed the hospital case records, and collected primary tumor tissue. Out of the 2,032 cases identified, 234 developed distant metastases after a median follow-up time of 2.7 years and had the first metastatic site documented (a total of 321 sites). Primary tumor microarray (TMA) cores were analyzed for 17 proteins using immunohistochemistry and for *erbB2 *using chromogenic *in situ *hybridization, and their associations with the first metastasis site were examined. The cancers were classified into luminal A, luminal B, HER2+ enriched, basal-like or non-expressor subtypes.

**Results:**

A total of 3,886 TMA cores were analyzed. Luminal A cancers had a propensity to give rise first to bone metastases, HER2-enriched cancers to liver and lung metastases, and basal type cancers to liver and brain metastases. Primary tumors that gave first rise to bone metastases expressed frequently estrogen receptor (ER) and SNAI1 (SNAIL) and rarely COX2 and HER2, tumors with first metastases in the liver expressed infrequently SNAI1, those with lung metastases expressed frequently the epidermal growth factor receptor (EGFR), cytokeratin-5 (CK5) and HER2, and infrequently progesterone receptor (PgR), tumors with early skin metastases expressed infrequently E-cadherin, and breast tumors with first metastases in the brain expressed nestin, prominin-1 and CK5 and infrequently ER and PgR.

**Conclusions:**

Breast tumor biological subtypes have a tendency to give rise to first distant metastases at certain body sites. Several primary tumor proteins were associated with homing of breast cancer cells.

## Introduction

Breast cancer is the most common cancer in women worldwide with approximately 1.15 million new cases diagnosed annually [1]. Although the prognosis of breast cancer patients is generally favorable and mortality has declined due to early detection and improved adjuvant therapies [2], distant metastases are not uncommon and women with advanced disease still have a median survival time of only approximately two years [3,4]. Bone, liver, lung and soft tissue metastases are common, but breast cancer can give rise to metastases at almost any site. The site of distant metastasis is associated with the length of survival; patients with brain metastases or metastases at multiple sites generally have the least favorable outcomes [4].

Besides host factors, the propensity of breast cancer to give rise to distant metastases depends on the molecular type of breast cancer, and the gene expression profile of the primary tumor may predict the risk of distant recurrence [5,6]. Breast cancers are often divided into five molecular subtypes based on their gene expression profiles (the luminal A, luminal B, HER2-enriched, basal-like, and the normal-like subtypes). These subtypes have preferential sites for distant relapse [7,8], and the subtype may also be associated with efficacy of systemic cancer therapies [9,10].

The gene array-based breast cancer molecular subtypes have been approximated using a panel of five surrogate markers (estrogen receptor (ER), progesterone receptor (PgR), human epidermal growth factor receptor-2 (HER2), epidermal growth factor receptor (EGFR), and cytokeratin-5 (CK5)) as assessed by immunohistochemistry [11]. Such immunohistochemistry-based subtypes have roughly similar clinical characteristics and behavior as the respective subtypes defined by gene expression arrays [11], and they also predict for the sites of distant metastases [12].

Several studies have addressed the relations between the primary breast tumor protein or mRNA expression profiles and the site of distant metastases [8,12-15], but these reports have rarely differentiated between the first and the later metastatic sites. During breast cancer progression distant metastases are found at an increasing number of different body sites [16], and due to cancer heterogeneity the associations between the primary tumor properties and the metastasis predilection sites may be increasingly confounded as cancer progresses. Therefore, in the present study we made an attempt to investigate the associations between the primary tumor protein expression, as assessed by immunohistochemistry, and the first site of cancer distant recurrence. We correlated the first distant metastatic site with 18 selected primary tumor molecular features that are linked with the breast cancer molecular subtype, epithelial-mesenchymal transition, myoepithelial or stem cell-like features, or are a potential drug target. To reduce the risk of a selection bias, the study is based on a nationwide cohort of breast cancer patients. The results suggest that the primary tumor protein expression pattern is often predictive for the first site of distant metastases.

## Materials and methods

### Patients

Women diagnosed with breast cancer and who lived within one of five well-defined geographical areas in Finland in 1991 or 1992 were identified from the files of the Finnish Cancer Registry [17]. This cohort includes 2,930 breast cancer patients comprising 53% of all breast cancers diagnosed in Finland in 1991 and 1992 (*n *= 5,551). Clinical data were extracted from the hospital case records and survival data were obtained also from the files of the Finnish Cancer Registry and the Statistics Finland.

**Figure 1:**
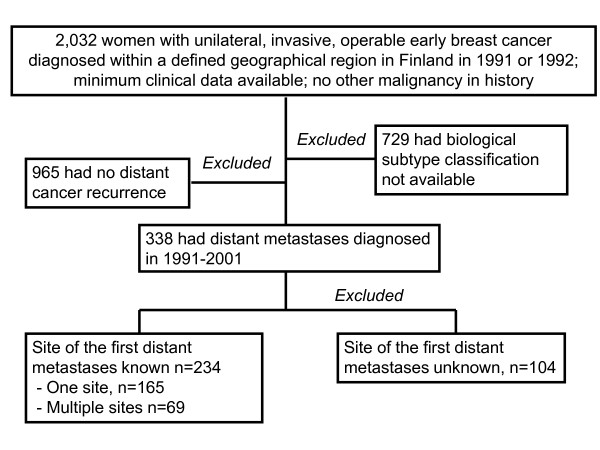
**Patient inclusion in the study**.

In each case an attempt to collect data on 50 characteristics was made. The minimum data for inclusion in the current study consisted of the date of the diagnosis, age at diagnosis, postsurgical Tumor-Node-Metastasis classification, availability of follow-up data after breast surgery and the vital status data at the end of follow up. We excluded from the study women who had distant metastases at the time of the diagnosis, bilateral breast cancer or other malignancy than breast cancer in history except basal cell carcinoma of the skin, cervical carcinoma *in situ*, or ductal or lobular breast carcinoma *in situ. *A total of 2,032 patients fulfilled these inclusion criteria (Figure 1). We next excluded patients whose breast tumor subtype classification could not be done because of missing information regarding expression of EGFR, HER2, ER, PgR, or CK5, or whose cancer did not give rise to distant metastases during the follow up (*n *= 1,694). In 104 cases of the remaining 338 patients with distant breast cancer recurrence, the first site of distant metastasis was not available leaving 234 (11.5%) of the 2,032 patients in the final study cohort. Of these 234 patients with a recorded first site of distant relapse, 165 (70.5%) were diagnosed with distant metastases at only one body site (considering the skeleton, the lungs and the subcutaneous tissue as one site), and 69 (29.5%) patients had first metastases at more than one site (median, 2.3 sites). The total number of the first metastatic sites in these 234 patients was 321.

A total of 137 (58.5%) of the 234 patients were treated with systemic adjuvant therapy after breast surgery. Adjuvant therapy consisted of tamoxifen in 53 cases, toremifene in 14, other hormonal therapy in three, CMF (cyclophosphamide, methotrexate, fluorouracil) in 56, and CEF (cyclophosphamide, epirubicin, fluorouracil) of CAF (cyclophosphamide, doxorubicin, fluorouracil) in five; in six cases the type of systemic adjuvant therapy given was not available.

The median follow-up time of the 234 subjects included in study was 9.3 years after the diagnosis to the date of death, and the median time to first detection of distant metastases from the date of breast cancer diagnosis was 2.7 years (range, from 0.1 to 9.6 years). The patients were usually followed up at hospital out-patient departments or at health centers according to the institutional practice. Besides bi-annual mammography, imaging examinations were usually not carried out in asymptomatic patients. Presence of metastatic breast cancer was confirmed histologically, cytologically, or by radiological imaging. The staging examinations at the time of detection of distant metastases typically involved an isotope bone scan and computed tomography of the trunk.

### Tumor tissue microarray and molecular subtypes

Permission to collect data from registries, files, and hospital records and to use formalin-fixed paraffin-embedded tissues for research purposes was provided by the Ministry of Social Affairs and Health, Finland (permission 123/08/97). Representative tumor regions were identified from H&E-stained formalin-fixed paraffin-embedded tissue sections, following which tissue microarray (TMA) blocks were constructed using a 0.6 mm diameter punch needle [18]. Sections of 5 μm were cut and processed for immunohistochemistry.

The cancers were classified into five biological subtypes; luminal A (ER+ and/or PgR+, HER2-), luminal B (ER+ and/or PgR+, HER2+), basal-like (ER-, PgR-, HER2-, CK5+ and/or EGFR+), HER2-enriched (HER2+, ER-, PgR-), and non-expressor type (negative for all five key classifiers). The non-expressor type corresponds to unclassifiable triple-negative breast cancer [19].

### Immunohistochemistry

Immunostainings for ER, PgR, HER2, EGFR, CK5, CK18, Ki-67, p53, GATA3, COX2, smooth muscle actin (SMA), and KIT were carried out using the antibodies and dilutions provided in Table 1. Immunostaining and evaluation of protein expression were carried out as described in detail elsewhere [18,20-22]. In brief, ER, PgR, CK5, CK18, Ki-67, p53, GATA3, and SMA expression were classified based on the percentage of positive tumor cells out of all tumor cells in the sample. Expression was considered negative when less than 10% of tumor cell nuclei expressed protein, except for Ki-67 and p53, where we used a cut-off of 20% [18,23]. Immunostainings for EGFR and KIT were classified based on the staining intensity accounting both for membranous and cytoplasmic staining rather than the percentage of staining cancer cells due to the uniform staining pattern of tumor, and using a scale negative (-), low (+), intermediate (++), or high (+++) [11,21]. Immunostaining for COX2 was considered positive when moderate to strong cytoplasmic staining was present in more than 10% of tumor cells, and immunostaining for HER2 was considered positive when strong intensity of membranous staining was present in the majority (> 50%) of cancer cells [18,22,24].

**Table 1:** Antibodies and their dilutions used in immunohistochemistry

Target	Clone	Dilution	Manufacturer
CK5	M7237	1:25	DakoCytomation, Glostrup, Denmark
CK18	NCL-CK18	1:20	Novocastra Laboratories Ltd, UK
COX2	160122	1:200	Cayman Chemical, Ann Arbor, MI, USA
E-cadherin	#4065	1:200	Cell Signaling Technology Inc, Danvers, MA, USA
EGFR	NCL-EGFR	1:500	Novocastra Laboratories Ltd
ER	6F11	1:500	Novocastra Laboratories Ltd
GATA3	HG3-31	1:300	Santa Cruz Biotechnology Inc, Santa Cruz, CA, USA
HER2	CB11	1:200	Novocastra Laboratories Ltd
Ki-67	MM-1	1:1000	Novocastra Laboratories Ltd
KIT	A4502	1:300	DakoCytomation
Nestin	MAB5326	1:400	Chemicon International Inc, Temecula, CA, USA
p53	D07	1:500	Novocastra Laboratories Ltd
PgR	312	1:500	Novocastra Laboratories Ltd
Prominin	AC133	1:10	Miltenyi Biotec GmbH, Germany
SMA	M0581	1:100	DakoCytomation
SNAI1	ARP33314	1:400	Aviva Systems Biology, San Diego, CA, USA
SNAI2	D-19	1:100	Santa Cruz Biotechnology Inc.

CK5, cytokeratin-5; CK18, cytokeratin 18; COX2, cyclooxygenase 2; EGFR, epidermal growth factor receptor; ER, estrogen receptor; GATA3, GATA binding protein 3; HER2, human epidermal growth factor receptor-2; PgR, progesterone receptor; SMA, smooth muscle actin.

To assess expression of SNAI1 (SNAIL), SNAI2 (SLUG), E-cadherin, prominin-1 (CD133/1) and nestin, the tissue sections were deparaffinized and rehydrated, and prior to immunostainings antigen retrieval was carried out in sodium citrate buffer (pH 6.0, 10 mmol/L) by heating the samples in an autoclave at 120°C for two minutes except for immunostaining for nestin, where no antigen retrieval was used. The immunostainings were performed following the manufacturer's protocol using the antibodies provided in Table 1 and a PowerVision+ Poly-HRP-histostaining kit (DVPB+110DAP, Technologies Co, Daly City, CA, USA) or a Vectastain-Elite goat kit (Vector Laboratories, Inc, Burlingame, CA, USA) to detect SLUG expression. The immunostainings were first graded as either negative (-), low expression (+, ≤ 10% of cells or nuclei positive), moderate expression (++, 11 to 50% positive), or high expression (+++, > 50% positive), but in further analysis expression was grouped as either negative (-) or positive (+, ++, or +++).

### Chromogenic *in situ *hybridization

The *HER2 *gene copy numbers were determined using chromogenic *in situ *hybridization (CISH) [18]. *HER2 *amplification was considered to be present when six or more signals were detected per nucleus in more than 50% of cancer cells or when signal clusters were seen.

### Statistical analyses

Frequency tables were analyzed using the chi squared test or Fisher's exact test. All *P *values are two-tailed.

## Results

### Cancer molecular subtype and the first site of distant metastases

A total of 3,886 representative tumor tissue samples were analyzed using immunohistochemistry with 17 different antibodies or with CISH. The most common first sites of distant metastases were the bone (39.6%), the liver (17.8%), and the lung (13.1%, Table 2).

**Table 2:** Breast cancer molecular subtype and the first site of distant metastases

					First distant site of breast cancer recurrence					
Breast cancer molecular subtype	No. of patients^1^	No. of metastatic sites	Bone	Liver	Lung	Non-regional lymph nodes	Skin	Pleura	Brain	Other
	n	n	n (%)	n (%)	n (%)	n (%)	n (%)	n (%)	n (%)	n (%)
Luminal A	123	164	77 (47.0)	29 (17.7)	14 (8.5)	10 (6.1)	12 (7.3)	9 (5.6)	3 (1.8)	10 (6.1)
Luminal B	30	43	15 (34.9)	5 (11.6)	7 (16.3)	5 (11.6)	5 (11.6)	3 (7.0)	0 (0.0)	3 (2.3)
HER2+/HR-	36	48	14 (29.2)	13 (27.1)	11 (22.9)	4 (8.3)	4 (8.3)	0 (0.0)	1 (2.1)	1 (2.1)
Basal-like	26	42	12 (28.6)	4 (9.5)	10 (20.8)	5 (11.9)	3 (7.1)	3 (7.1)	4 (9.5)	1 (2.4)
Non-expressor	19	24	9 (37.5)	6 (25.0)	0 (8.3)	2 (8.3)	0 (0.0)	3 (12.5)	3 (12.5)	1 (4.2)
Total	234	321	127 (39.6)	57 (17.8)	42 (13.1)	26 (8.1)	24 (7.5)	18 (5.6)	11 (3.4)	16 (5.0)

^1^41, 13, 12, 16 and five patients with luminal A, luminal B, HER2+/HR-, basal-like, and non-expressor type of breast cancer, respectively, had more than one first metastatic site. HER2, human epidermal growth factor receptor-2; HR, hormone receptor.

Women with luminal A cancer had the first metastatic site in the bone more often compared with women with breast cancer of some other subtype (in 77 (47.0%) of 164 vs. 50 (31.8%) of 157 first distant sites the site was in the bone, *P *= 0.006, Table 2). HER2-positive and ER- and PgR-negative cancers tended to give rise first to liver metastases more frequently compared with the other subtypes (in 13 (27.1%) of 48 vs. 44 (16.1%) of 273 first distant sites the site was in the liver, *P *= 0.067). Compared with the other biological subtypes, luminal A cancers rarely gave rise to lung metastases first (in 14 (8.5%) of 164 cases vs. 28 (17.8%) of 157 cases the first distant site was the lung, respectively, *P *= 0.014). Brain metastases were rarely the first metastatic site (3.4%), and brain metastases occurred more frequently in women who had either the basal type or the non-expressor type as compared with the rest of the subtypes (in 4 (9.5%) of 42 vs. 7 (2.5%) of 279 sites, *P *= 0.043; and in 3 (12.5%) of 24 vs. 8 (2.8%) out of 297 sites, *P *= 0.04, respectively). First metastases manifested rarely in the ovary or in the peritoneum (*n *= 9); this occurred only in women diagnosed with hormone receptor (HR)-positive cancer.

**Figure 2:**
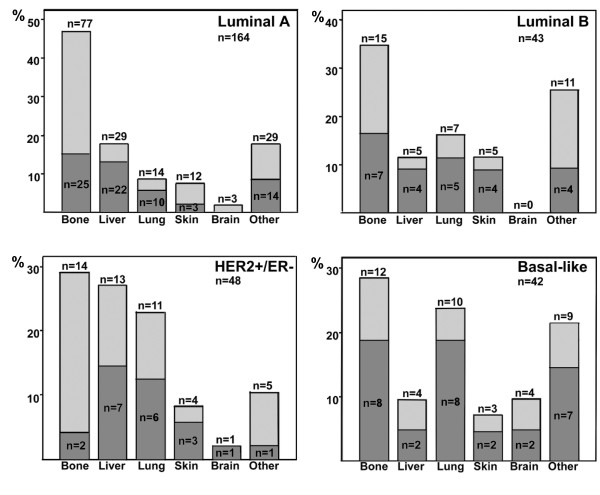
**Associations between four biological subtypes of breast cancer and the first sites of distant metastases**. The dark-shaded (lower) parts of the columns depict the proportion of cancers that gave rise to more than one first metastatic site. The total number of metastatic sites is provided in each panel. ER, estrogen receptor; HER2, human epidermal growth factor receptor-2.

Women with the basal type of breast cancer had more often the first distant metastases at multiple sites compared with women with other subtypes of breast cancer (13 (50.0%) of 26 vs. 56 (26.9%) of 208, *P *= 0.015; Figure 2).

### Tumor protein expression and the first site of distant metastases

**Figure 3:**
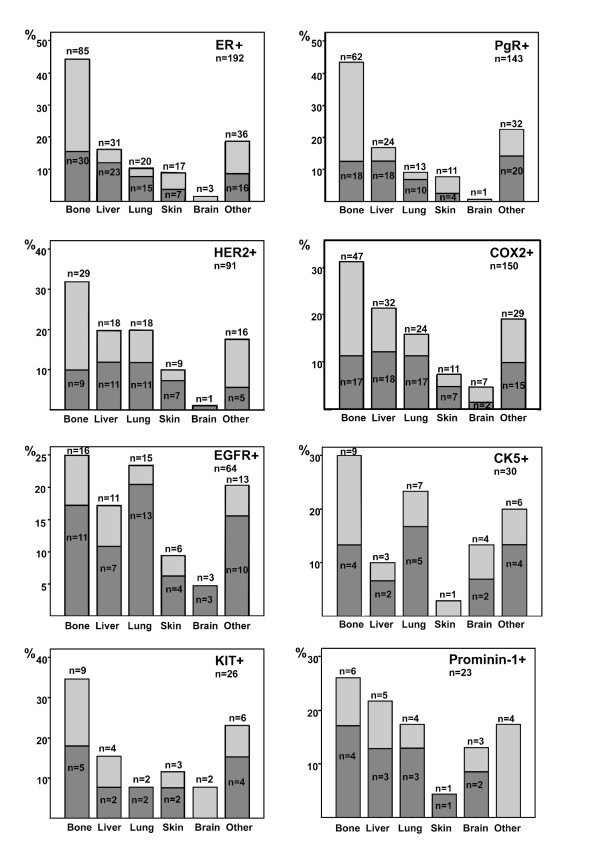
**Association of eight breast tumor proteins with the first sites of distant metastases**. The dark-shaded (lower) parts of the columns depict the proportion of cancers that gave rise to more than one first metastatic site. The total number of metastatic sites is provided in each panel. CK5, cytokeratin-5; COX2, cyclooxygenase 2; EGFR, epidermal growth factor receptor; ER, estrogen receptor; HER2, human epidermal growth factor receptor-2; PgR, progesterone receptor.

Expression of several of the 17 single proteins analyzed from the breast tumor was associated with the first site of cancer distant recurrence. The associations between expression of eight breast tumor proteins and the first distant metastasis sites are provided in Figure 3. Women whose breast cancer expressed EGFR, in particular, had frequently more than one metastatic site at the time when the first distant metastases were detected.

Bone metastases were often the first distant metastasis site when the tumor expressed SNAI1 or ER (87 (60.0%) of the 145 patients with SNAI1-positive tumor were first diagnosed with bone metastases as compared with 29 (42.0%) of the 69 women with SNAI1-negative tumor, *P *= 0.014; similarly, 85 (59.9%) of the 142 women with ER-positive cancer compared with 42 (46.7%) of the 90 women with ER-negative tumor had the first distant metastases in the bone, *P *= 0.049, Table 3). On the other hand, breast cancers that expressed COX2 were infrequently associated with first distant metastases in the skeleton (47 (43.5%) of the 108 COX2-positive cancers vs. 79 (63.7%) of the 124 COX2-negative cancers, *P *= 0.002). Similarly, women with HER2-positive cancer infrequently had bone as the first metastatic site compared with those whose cancer was HER2-negative (29 (43.9%) of 66 vs. 98 (58.3%) of 168, respectively, *P *= 0.047).

**Table 3:** Breast tumor protein expression and the first site of distant recurrence

				First distant site of breast cancer recurrence				
Protein	No. of breast cancers with protein expression/No. of breast cancers examined	No. of first metastatic sites from cancers with protein expression/No. of all first metastatic sites^a^	Bone	Liver	Lung	Skin	Brain	Other
	n/N^b ^(%)	n/N (%)	n/N^c ^(%)	n/N^c ^(%)	n/N^c ^(%)	n/N^c ^(%)	n/N^c ^(%)	n/N^c ^(%)
COX2	108/232 (46.6)	150/319 (47.0)	47/108 (43.5)***	24/108 (22.2)	32/108 (29.6)*	11/108 (10.2)	7/108 (6.5)	29/108 (26.9)
HER2	66/234 (28.7)	91/321 (28.3)	29/66 (43.9)**	18/66 (27.3)	18/66 (27.3)**	9/66 (13.)	1/66 (1.5)	16/66 (24.2)
ER	142/232 (61.2)	192/317 (60.6)	85/142 (59.9)**	31/142 (21.8)	20/142 (14.1)*	17/142 (12.0)	3/142 (2.1)**	36/142 (25.4)
PgR	106/230 (46.1)	143/316 (45.3)	62/106 (58.5)	24/106 (22.6)	13/106 (12.3)**	11/106 (10.4)	1/106 (0.9)**	32/106 (30.2)
EGFR	36/196 (18.4)	64/269 (23.8)	16/36 (44.4)	11/36 (30.6)	15/36 (41.7)****	6/36 (16.7)	3/36 (8.3)	13/36 (36.1)
CK5	21/207 (10.1)	30/285 (10.5)	9/21 (42.9)	3/21 (14.3)	7/21 (33.3)**	1/21 (4.8)	4/21 (19.0)**	6/21 (28.6)
Nestin	19/206 (9.2)	27/284 (9.5)	10/19 (52.6)	3/19 (15.8)	4/19 (21.1)	2/19 (10.5)	3/19 (15.8)*	5/19 (26.3)
Prominin-1	15/180 (8.3)	23/246 (9.3)	6/15 (40.0)	3/15 (20.0)	4/15 (26.7)	1/15 (6.7)	3/15 (20.0)**	6/15 (40.0)
SMA	9/219 (4.1)	13/300 (4.3)	4/9 (44.4)	2/9 (22.2)	2/9 (22.2)	0/9 (0.0)	2/9 (22.2)*	3/9 (33.3)
SNAI1	145/214 (67.8)	200/296 (67.6)	87/145 (60.0)**	28/145 (19.3)***	22/145 (15.2)	17/145 (11.7)	5/145 (3.4)	41/145 (28.3)
SNAI2	59/222 (26.6)	81/304 (26.6)	33/59 (55.9)	13/59 (22.0)	13/59 (22.0)	3/59 (5.1)*	3/59 (5.1)	16/59 (27.1)
CK18	215/222 (96.8)	294/303 (97.0)	114/215 (53.0)	53/215 (24.7)	39/215 (18.1)	22/215 (10.2)	10/215 (4.7)	56/215 (26.0)
E-cadherin	189/211 (89.6)	259/288 (89.9)	104/189 (55.0)	46/189 (24.3)	34/189 (18.0)	16/189 (8.7)*	8/189 (4.2)	51/189 (27.0)
KIT	17/210 (8.1)	26/291 (8.9)	9/17 (52.9)	4/17 (23.5)	2/17 (11.8)	3/17 (17.6)	2/17 (11.8)	6/17 (35.3)
GATA3	151/219 (68.9)	209/302 (69.2)	88/151 (58.3)	38/151(25.2)	21/151 (13.9)*	16/151 (10.6)	5/151 (3.3)	41/151 (27.2)
Ki67	112/220 (50.9)	154/299 (51.5)	59/112 (52.7)	30/112(26.8)	24/112 (21.4)	9/112 (8.0)	5/112 (4.7)	27/112 (24.1)
p53	57/213 (26.8)	86/292 (29.5)	30/57 (52.6)	18/57 (31.6)	15/57 (26.3)*	5/57 (8.8)	3/57 (5.3)	15/57 (26.3)

^a^One breast cancer may have given rise to more than one first metastatic sites.

^b^Two to 54 tumors were not analyzed due to lack of tissue material.

^c^The number of first metastatic sites from cancers with the protein expression/the number of breast cancers that expressed the protein.

*P < 0.10, **P < 0.05, ***P < 0.01, ****P < 0.001.

CK5, cytokeratin-5; CK18, cytokeratin 18; COX2, cyclooxygenase 2; EGFR, epidermal growth factor receptor; ER, estrogen receptor; GATA3, GATA binding protein 3; HER2, human epidermal growth factor receptor-2; PgR, progesterone receptor; SMA, smooth muscle actin.

Detection of the first distant recurrence in the liver was associated with absence of SNAI1 expression in the breast tumor. The liver was the first distant metastatic site in 28 (19.3%) of the 145 women with SNAI1-positive cancer as compared with 25 (36.2%) of the 69 patients whose breast tumor did not express SNAI1 (*P *= 0.007).

Lung metastases as the first distant site were strongly associated with breast tumor EGFR expression. Of the 36 women who had breast cancer expressing EGFR, 15 (41.7%) were first diagnosed with lung metastases as compared with 20 (12.5%) of the 160 women whose cancer was EGFR-negative (*P *< 0.0001). HER2-positive, CK5-positive, p53-positive, and COX2-positive breast cancers also often gave rise first to lung metastases (18 (27.3%) of 66 vs. 24 (14.3%) of 168, *P *= 0.020; 7 (33.3%) of 21 vs. 29 (15.3%) of 189, *P *= 0.042; 15 (26.3%) of 57 vs. 25 (16.0%) of 156, *P *= 0.089; and 32 (29.6%) of 108 vs. 25 (20.2%) of 124, *P *= 0.095, respectively). Women who had ER-positive, PgR-positive, or GATA3-positive cancer were infrequently diagnosed first with lung metastases (20 (14.1%) of 142 vs. 21 (23.3%) of 90, *P *= 0.072; 13 (12.3%) of 106 vs. 29 (23.4%) of 124, *P *= 0.030; and 21 (13.9%) of 151 vs. 16 (23.5%) of 68, *P *= 0.079, respectively).

Most breast cancers expressed E-cadherin in the series (189 (89.6%) of the 211 evaluable cancers). Absence of E-cadherin and SNAI2 expression in the breast tumor tended to be associated with the first distant metastases in the skin (or the subcutaneous tissue). Only 16 (8.5%) of the 189 women whose breast cancer expressed E-cadherin had the first distant metastases in the skin compared with 5 (22.7%) of the 22 women whose cancer was E-cadherin-negative (*P *= 0.051). Three (5.2%) of the 59 women whose breast cancer expressed SNAI2 had the first distant cancer recurrence in the skin compared with 21 (12.9%) of the 163 women whose cancer did not express SNAI2 (*P *= 0.098). Many E-cadherin-negative tumors had lobular morphology (11 (50.0%) of 22).

Brain metastases were rarely the first site of distant recurrence. However, they were associated with expression of some of the proteins examined. Nestin, prominin-1, CK5, and SMA was more often expressed in the primary breast tumor when the first metastases were found in the brain compared with other first metastatic sites (3 (15.8%) of 19 vs. 7 (3.7%) of 187, *P *= 0.053; 3 (20.0%) of 15 vs. 6 (3.6%) of 165, *P *= 0.029; 4 (19.0%) of 21 vs. 7 (3.8%) of 186, *P *= 0.016; and 2 (22.2%) of 9 vs. 9 (4.3%) of 210, *P *= 0.068, respectively). Women who had ER-positive or PgR-positive cancer rarely had the brain as the first distant metastatic site compared with women whose cancer did not express these proteins (3 (2.1%) of 142 vs. 8 (8.9%) of 90, *P *= 0.025; 1 (0.9%) of 106 vs. 10 (8.1%) of 124, *P *= 0.012).

### *HER2 *amplification and the first site of distant recurrence

Presence of *HER2 *amplification in CISH in the breast tumor and tumor HER2 expression in immunohistochemistry were closely associated; 141 (95.2%) of the 148 breast tumors that did not contain *HER2 *amplification did not express HER2, whereas 57 (80.3%) of the 71 tumors with *HER2 *amplification expressed HER2 protein (*P *< 0.0001). In line with the results obtained with immunohistochemistry, patients who had *HER2 *amplification in the breast tumor had more often the first distant metastases detected in the lung as compared with women whose cancer was *HER2*-negative (20 (28.2%) of 71 vs. 20 (13.5%) of 148, *P *= 0.010), whereas *HER2*-amplified breast cancers seldom gave first rise to bone metastases compared with *HER2*-negative cancers (32 (45.1%) of 71 vs. 90 (60.8%) of 148, *P *= 0.028).

### The first site of distant recurrence and presence of common drug targets

Finally, we examined whether the site of the first distant cancer recurrence could be used as a surrogate for predicting presence of common drug target proteins in the primary tumor. We selected for this analysis those tumors that had first given rise to metastases either in the bone (*n *= 102), the liver (*n *= 48), or the lung (*n *= 33) and that could be studied for expression of the potential therapeutic target proteins ER, PgR, HER2, and EGFR (Table 4). Tumors that gave first rise to bone metastases were frequently ER positive and/or PgR positive (67.6%) and rarely EGFR-positive (15.7%), whereas breast cancers that gave first rise to lung metastases often expressed HER2 (48.5%) and EGFR (45.5%), very frequently expressed either HER2 or EGFR (75.8%), and infrequently expressed PgR (27.3%). Breast cancers that gave rise first to liver metastases frequently expressed ER (52.1%), PgR (37.5%), or HER2 (33.3%).

**Table 4:** Breast tumor ER, PgR, HER2 and EGFR expression and the bone, the liver and the lungs as the first sites of distant recurrence

Primary tumor protein expression	First distant site of breast cancer recurrence		
	Bone	Liver	Lung
	*N *= 102 n (%)	*N *= 48 n (%)	*N *= 33 n (%)
ER +	64 (62.7)	25 (52.1)	15 (45.5)
PgR +	46 (45.1)	18 (37.5)	9 (27.3)
ER + and/or PgR +	69 (67.6)	26 (54.2)	15 (45.5)
HER2 +	26 (25.5)	16 (33.3)	16 (48.5)
EGFR + (HER1)	16 (15.7)	11 (22.9)	15 (45.5)
EGFR + and/or HER2 +	37 (36.3)	20 (41.7)	25 (75.8)

EGFR, epidermal growth factor receptor; ER, estrogen receptor, HER1, human epidermal growth factor receptor-1; HER2, human epidermal growth factor receptor-2; PgR, progesterone receptor.

## Discussion

The present results suggest that the first sites of breast cancer distant metastases reflect expression of the primary breast tumor proteins, and that different biological subtypes of breast cancer have a propensity to give rise to distant metastases at specific body sites. Breast cancer is a heterogeneous disease, and, as expected, the associations detected were only partial. Yet, the current results lend support to the hypothesis that the first site of distant metastasis may give valuable clues of the biology of the disease and perhaps also for selection of therapy.

In agreement with some prior studies, we found that ER-positive breast cancers had a propensity to give rise to bone metastases and that HER2-positive cancers give rise to bone metastases less frequently than ER-positive tumors [12,13,24]. As a novel finding, we found that SNAI1-positive cancers also tend to home into the bone, whereas COX2-positive cancers do not. SNAI1 is a zinc finger transcriptional repressor of *CDH1*, which encodes E-cadherin. Downregulation of E-cadherin activates the "epithelial-mesenchymal transition", which is crucial for the dissemination and invasion of cancer cells, loss of epithelial differentiation and acquisition of a mesenchymal phenotype [25], which might augment breast cancer metastasis into the bone. Moderate to strong (elevated) expression of COX-2 protein is observed in approximately one third of early breast cancers, and COX-2 expression is associated with a large tumor size, a high histological grade, a negative HR status, a high cell proliferation rate, high p53 expression, presence of HER-2 amplification, and unfavorable survival [22]. In accordance with the present data, some observational findings suggest that the use of COX-2 inhibitors could reduce the risk of bone metastases in stage II and III breast cancer [26], but this hypothesis awaits confirmation in clinical trials.

The strongest single association detected in the present study was the one between the breast primary tumor EGFR expression and lung metastases, and as many as 75.8% of all those patients whose first distant recurrence was in the lung had either EGFR-positive or HER2-positive primary breast tumor (Table 4). Experimental mouse models suggest that EGFR is important for tumor cell motility and invasion, and HER2 for tumor cell intravasation [27]. The EGFR ligand epiregulin, COX2, and the matrix metalloproteinases 1 and 2 collectively facilitate tumor cell entry into the bloodstream and the breaching of lung capillaries by circulating tumor cells to seed lung metastases [28]. In line with these and the present findings, erlotinib, an orally administered inhibitor of the EGFR, prevented development of pulmonary metastases in a spontaneous pulmonary metastasis breast cancer mouse model, where expression of EGF and EGFR turned out to be strong in pulmonary metastatic nodules compared with primary breast cancer [29]. Hypothetically, clinical testing of dual inhibitors of EGFR and HER2, such as lapatinib, neratinib, and BIBW 2992 (afatinib), might thus be of particular interest in the relatively small subset of breast cancer patients whose disease first recurs in the lungs.

In keeping with other studies [16], brain metastases were infrequent as the first site of distant recurrence in the present series. Brain metastases have been found to be associated with HR-negative and triple-negative (ER-, PgR-, HER2-) breast cancers [8,12,14,15,24,30], EGFR expression [15], low Bcl-2 [15], and breast cancer HER2 expression and presence of *HER2 *amplification [12,16,31]. However, one large study examining patients from the pre-trastuzumab era did not find breast tumor HER2 expression to be associated with brain metastases [15], and a recent study found a higher incidence of brain metastases only in those breast cancer patients with HER2-positive breast cancer who had been treated with trastuzumab [32]. In autopsy series, central nervous system (CNS) metastases can be detected in approximately 30% of patients who have died from breast cancer [33,34], and since trastuzumab penetrates poorly into the CNS but controls effectively non-CNS disease, this might lead to manifestation of brain metastases during trastuzumab treatment in some patients. The patients in the present series were treated before the trastuzumab era.

Brain metastases as the first site of distant recurrence were associated most frequently with the basal subtype and primary tumor expression of nestin, prominin-1, or CK-5, and, on the other hand, they were rare when the breast tumor expressed ER or PgR. To our knowledge, the association between breast primary tumor nestin and prominin-1 expression and the first recurrence of cancer in the brain has not been reported earlier. Nestin is a type VI intermediate filament protein that is expressed in nerve cells and is considered a marker for neural stem cells [35], and prominin-1/CD133 is a neural and hematopoietic stem cell marker [36]. In a landmark study, CD133-positive cell subpopulation isolated from human brain tumors exhibited stem cell properties and turned out to be the brain tumor initiating cells in a mouse model [37]. The biological mechanisms associated with these proteins and breast cancer brain metastases remain hypothetical, but breast cancer cells that express CD133 or nestin might share features of neural stem cells and might thus be particularly well adapted to the brain microenvironment to initiate brain metastases.

This retrospective study has a few limitations. We did not have histological tissue available from the first distant metastases. It is now established that a biopsy from a metastatic site may yield a different result regarding the ER or the HER2 status compared with the primary tumor in 5 to 30% of cases [38,39]. This likely reflects both heterogeneity of the disease already in the primary tumor [40] and cancer progression with time, although the biological type of the primary tumor is largely maintained in distant metastases [34]. The concept of the first site of distant metastasis is not well-defined, because at autopsy breast cancer metastases can be found in many organs [34], and likely more metastatic sites are found at the time of detection of the first metastases the closer the patient is examined. Classification of the primary tumors into categories based on protein expression in immunohistochemistry is somewhat subjective, and the optimal cut-offs selected are still being discussed even for proteins such as ER [41]. The adjuvant systemic treatments administered to 58.5% of the patients may have influenced the first site of distant metastases, but adjuvant treatment has been considered mandatory in the management of high-risk early breast cancer for a few decades. We made an attempt to avoid a selection bias by using a nationwide series as the basis of the study, but a relatively large proportion of patients who developed distant metastases had to be excluded due to absence of adequate tissue material or data on the first metastatic site. Finally, due to multiple proteins analyzed, many statistical analyses were carried out, which increases the probability of obtaining a significant association by chance. Due to these limitations and confounders, the study results need to be viewed with some caution, although several of the findings are well in line with prior clinical studies or with experimental data.

## Conclusions

We conclude that breast cancer biological subtypes are associated with the first site of distant recurrence, and that many commonly assessed proteins are associated with homing of breast cancer at distant sites. In line with prior studies, we found that ER-positive breast cancers often home in the bone, but we report several novel findings. Besides ER, bone metastases are frequently associated with tumor SNAI1 expression. Breast cancers that first give rise to lung metastases very often express either EGFR or HER2, which suggests that such cancers might be a potential target group for dual HER1 and HER2 inhibitors, such as lapatinib or neratinib. Cancers that express the basal CK-5 or the cell stemness-linked proteins prominin-1 or nestin are associated with the brain as the first site of distant recurrence.

## Abbreviations

CISH: chromogenic *in situ *hybridization; CK5: cytokeratin-5; CNS: central nervous system; EGFR: epidermal growth factor receptor; ER: estrogen receptor; HER2: human epidermal growth factor receptor-2; H&E: hematoxylin and eosin; HR: hormone receptor; PgR: progesterone receptor; SMA: smooth muscle actin; TMA: tumor microarray.

## Competing interests

The authors declare that they have no competing interests.

## Authors' contributions

HS and HJ designed the study, performed the statistical analyses, drafted the manuscript, and performed the immunohistochemical analysis. JL performed collection of the tumor samples and clinical data and performed the statistical analyses. ML, TL, KH, LS, VK, and TTH performed collection of the tumor samples and clinical data. AR and PH performed the immunohistochemical analysis.

JI performed the immunohistochemical analysis and performed collection of the tumor samples and clinical data. All authors critically revised the manuscript and approved its final form.
